# The secret of youth: how is systemic rejuvenation achieved at the single cell level?

**DOI:** 10.1093/lifemedi/lnac018

**Published:** 2022-06-28

**Authors:** Lingna Wang, Jiaqing Liu, Huicong Liu, Masayuki Yazawa, Fangfang Zhu

**Affiliations:** School of Biomedical Engineering, Shanghai Jiao Tong University, Shanghai 200030, China; School of Biomedical Engineering, Shanghai Jiao Tong University, Shanghai 200030, China; School of Biomedical Engineering, Shanghai Jiao Tong University, Shanghai 200030, China; Department of Rehabilitation and Regenerative Medicine, Columbia University, New York, NY 10032, USA; Columbia Stem Cell Initiative, Columbia University, New York, NY 10032, USA; Department of Pharmacology, Columbia University, New York, NY 10032, USA; School of Biomedical Engineering, Shanghai Jiao Tong University, Shanghai 200030, China

Heterochronic parabiosis (HP) refers to a condition in which two animals of different ages are surgically connected to share a common circulatory system. With the formation of microvessels at the connection site, the aged individual receives blood-borne factors from young individual, and vice versa, the young individual receives blood-borne factors from aged individual. Therefore, HP provides an opportunity to investigate how and what blood-borne factors could alter the health conditions in both young and more importantly aged organisms. In addition, the identification of rejuvenating factors in HP is a promising approach to delay the aging process and lead to therapeutic products to the reversal of aging or treatment of aging-related diseases.

**Figure 1.**
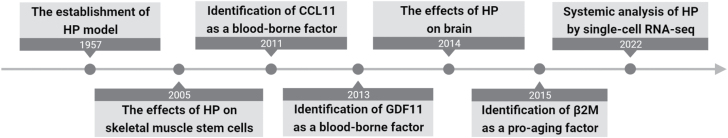
A graphical summary of the history of HP model and identification of blood-borne factors.

The development of HP has been seen as a great progress in the past century in the aging field and such advanced technologies have opened new avenue to address the long-lasting question of the molecular mechanism underlying aging and also identify new strategies for treating aging. First established in 1957 [1], the HP rodents have been served as a unique animal model to elucidate the effects of young and old blood on aging (Fig. 1). After that, aging-associated phenotype of skeletal muscle stem cells and hepatic cells were reported to be reversed in such model [2], providing proof of concept in rejuvenation of aged cells under the fused circulation system. Since then, the effects of HP on various tissues have been widely studied and to date, rejuvenative effects were reported in brain, vascular, and heart [2–5]. Meanwhile, several pro-aging or pro-rejuvenative factors have been identified in the circulation, such as growth and differentiation factor 11 (GDF11) [3, 4], C–C motif chemokine 11 (CCL11) [5] and β2 microglobulin (B2M) [6]. However, these studies have been focusing on only one or a few solid tissues, and the identified rejuvenating factors are only capable of reversing the aging-related phenotype in single cell types. As emerging studies have shown the immune system represents a key therapeutic target for slowing down aging [7], it remains largely unknown whether young blood rejuvenates aged hematopoietic stem and progenitor cells (HSPCs) that change the entire blood and immune system so as to establish a revified milieu. Similarly, it is also unclear whether there are potential systemic effects on stem cell maintenance and peripheral tissue/organ homeostasis conferred by the revitalized hematopoietic and immune system or blood-borne factors. As HSPCs and their differentiated blood and immune cell populations are highly heterogenous, and different cell types will likely be differently affected in HP, such an analysis calls for a higher resolution systemic approach.

**Figure 2.**
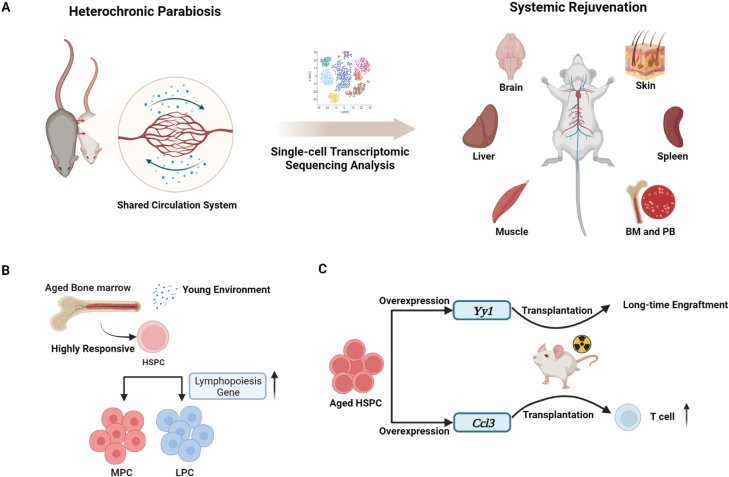
Systemic investigation of the effects of blood-borne factors in HP model. (A) Single-cell transcriptomic sequencing analysis of 7 tissues/organs from HP depicts a systemic atlas of the effects of young blood on aging. (B) Blood-borne factors in young mice reset regulatory programs that enhances lymphoid differentiation potential of HSPCs in aged mice. MPC, myeloid progenitor cell; LPC, lymphoid progenitor cell. (C) Overexpression of identified rejuvenating factor Yy1 in aged HSPCs promoted their long-term engraftment ability, and Ccl3 overexpression increased T lymphopoietic differentiation potential of aged HSPCs.

To address these questions, Ma and colleagues took advantage of single-cell RNA sequencing (scRNA-seq) for global transcriptomic profiling to reveal the cellular heterogeneity and molecular changes in multiple tissues/organs in HP (Fig. 2A) [8]. While the molecular physiology of aging is varied across cell types and tissues and the niche environment of different cells and their communications are also dramatically different, scRNA-seq in a systematic level can largely facilitate our understanding of the rejuvenation paradigm. Similarly, in a parallel work, Róbert Pálovics et al. also applied scRNA-seq in 20 organs across the entire organism in HP and showed that young blood reversed global gene expression loss and increased the expression of genes encoding components of the electron transport chain [9].

In the effort to analyze single-cell transcriptome across multiple tissues/organs in HP mice, including enriched HSPCs, bone marrow, peripheral blood, spleen, skin, liver, skeletal muscle and brain, Ma and colleagues acquired approximately 164 000 high-quality single-cell transcriptomes and annotated 108 major cell types for subsequent analyses. They focused on HSPCs because they found HSPCs are the most responsive to the changing environment (Fig. 2B). In HP, organismal exposure to young blood boosted lymphopoiesis program, rewired the continuum of HSPC differentiation to a younger state, and transcriptionally rescued hematopoietic stem cells (HSCs) compromised by aging, as manifested by the re-establishment of intercellular interactions through reactivation of cytokines and cytokine receptors upon exposure to young blood. Using the CD45 congenic mice to trace cell origins in the HP model, they also demonstrated that blood-borne factors from young mice rejuvenated HSPCs in the bone marrow of old mice while rejuvenation of the peripheral blood and spleen in aged mice is achieved mainly by replenishment of immune cells from young blood. The inclusion of experiments to distinguish aging/rejuvenation from cell crossover adds significant depth to this study, suggesting that, in HP, young mice could alter the hematopoietic and immune system of old mice through multiple mechanisms.

Aging-associated progressive decline in regenerative capacity is primarily decided by tissue-resident stem cells whose overall status, in turn, relies on both the stem cells themselves and the surrounding niche cells. Therefore, this group also investigated the effects of HP on other organs and tissues besides the hematopoietic and immune systems. Not surprisingly, they found that aging-related changes in tissue-resident stem cells, to varying degrees, were reversed extrinsically by systemic factors in young blood. This result not only points out the cell-type specific mechanisms for revitalization but also suggests that blood-borne factors can induce systemic reversal of aging as a goal that we have been eager to accomplish in human beings. However, in this study, only skin, liver, skeletal muscle, and brain were selected for analysis. Therefore, it remains to be explored whether HP could exhibit similar effects on other tissues and organs. If so, the next question would be whether they require the same or different rejuvenation factors.

In this study, Ma and colleagues revealed the underlying cellular mechanism of HP-mediated rejuvenation of aging systematically at the single cell level across multiple tissues. More importantly, they also identified candidate pro-youth factors that could be potentially applicable as intervention strategies for aging and aging-related diseases in the future. For example, their results suggest that chemokines and cytokines, such as Ccl3, Ccl4, Vegfa and Cxcl2, and Tnfaip3, Nfkbia and Nfkbiz, which belong to anti-inflammatory targets of the TNF-α pathway, are associated with HP-mediated rejuvenation of HSPCs. On the other hand, apoptosis- or senescence-associated secretory phenotype (SASP)-associated factors, such as the anti-inflammatory and NF-κB pathway inhibitory factor Gilz, were downregulated in old solid tissues upon exposure to young blood. Notably, introduction of Ccl3 in aged HSPCs enhanced their differentiation potential toward T cells in long-term engraftment (Fig. 2C). This outcome is exciting and meaningful as myeloid-biased differentiation is a hallmark of aged HSCs [10]. Therefore, these candidates and their functional validations add to the list of currently known rejuvenating factors and indicate that multiple factors may be necessary to result in the systemic aging reversal. Interestingly, chromatin organization and remodeling genes could not be rescued by exposure to young blood, while overexpression of Yy1, a key epigenetic regulator, enhanced the engraftment ability of aged mouse HSPCs (Fig. 2C), suggesting that aging memories are refractory to changing by environmental factors and requires other mechanism for rewriting.

In summary, Ma and colleagues have demonstrated how the young systemic milieu impacts the aged milieu and vice versa in the HP model. This comprehensive state-of-the-art resource reveals how blood-borne factors exert profound effects on systemic and global regulation of the hematopoietic and immune systems as well as tissue homeostasis. Thus, this work opens new vistas to further explore the mechanisms underlying the mysterious pro-youth effects of young blood and identifies blood-borne factors as potential candidates for systemic rejuvenation. As human lifespan becomes longer, the demand to reverse aging and aging-related diseases also becomes greater. Besides HP, research on developing new aging-related interventions is quickly emerging, such as caloric restriction, gut microbiota transfer, and supplementation of nicotinamide mononucleotide, while the underlying mechanisms of different interventions may be complex and different. Therefore, more in-depth investigations into and a combination of these different interventions will, hopefully, lead to a long-awaited success in anti-aging therapy.
